# Sex-Dependent Depression-Like Behavior Induced by Respiratory Administration of Aluminum Oxide Nanoparticles

**DOI:** 10.3390/ijerph121215011

**Published:** 2015-12-09

**Authors:** Xin Zhang, Yan Xu, Lian Zhou, Chengcheng Zhang, Qingtao Meng, Shenshen Wu, Shizhi Wang, Zhen Ding, Xiaodong Chen, Xiaobo Li, Rui Chen

**Affiliations:** 1 Key Laboratory of Environmental Medicine Engineering, Ministry of Education, School of Public Health, Southeast University, Nanjing 210009, China; zx_ekin@163.com (X.Z.); Zhangchengcheng2015@hotmail.com (C.Z.); mqtmengqingtao@hotmail.com (Q.M.); shenshenwu2015@hotmail.com (S.W.); shizhiwang2009@hotmail.com (S.W.); 2 Department of Environmental Health and Endemic Disease Control, Jiangsu Provincial, Center for Disease Prevention and Control, Nanjing 210009, China; cdcxy@vip.sina.com (Y.X.); jonneylian@163.com (L.Z.); jscdc@126.com (Z.D.); jscxd@126.com (X.C.)

**Keywords:** depressive, nanoparticles, glutamate, aluminum oxide, sex difference

## Abstract

Ultrafine aluminum oxide, which are abundant in ambient and involved occupational environments, are associated with neurobehavioral alterations. However, few studies have focused on the effect of sex differences following exposure to environmental Al_2_O_3_ ultrafine particles. In the present study, male and female mice were exposed to Al_2_O_3_ nanoparticles (NPs) through a respiratory route. Only the female mice showed depression-like behavior. Although no obvious pathological changes were observed in mice brain tissues, the neurotransmitter and voltage-gated ion channel related gene expression, as well as the small molecule metabolites in the cerebral cortex, were differentially modulated between male and female mice. Both mental disorder-involved gene expression levels and metabolomics analysis results strongly suggested that glutamate pathways were implicated in sex differentiation induced by Al_2_O_3_ NPs. Results demonstrated the potential mechanism of environmental ultrafine particle-induced depression-like behavior and the importance of sex dimorphism in the toxic research of environmental chemicals.

## 1. Introduction

Previous studies have indicated an association between adverse health effects and respiratory exposure to airborne ultrafine particulate matters with average aerodynamic diameter of up to 0.1 μm [1,2]. Given that the chemical components of particulate matters may vary with location, which may affect toxicity, the study of health associations of particle chemical constituents is critically required. Metals represent a portion of the elements found within airborne particulate matters (PM), which are important as they have high potential for chemical reactivity *in vivo* and *in vitro* [3]. Aluminum (Al) is one of the most consistent metal components of PM in the ambient environment [4,5] and in the Al_2_O_3_ form it is relatively stable. Ultrafine Al_2_O_3_ particles are widely used in insulator layers, powder coatings, and fluorescent lamp reflecting materials. According to the Chinese Health standard for dusts of aluminum, aluminum oxide, and aluminum alloys in the air of workplace (GB11726-89), the maximum allowable concentration for Al_2_O_3_ is 6 mg/m^3^. Among these forms nanoscale Al_2_O_3_ particles could suspend longer in air and be uptaken through the respiratory tract. These ultrafine particles differ from the same conventional material in terms of physical, chemical, and biological characteristics; therefore, they are more likely to be toxic compared with same conventional-sized materials [6]. Once entering circulation, environmental ultrafine particles could induce impairments in other vital organs, such as the central nervous system. Thus, increased and inevitable occupational and ambient environment exposures to ultrafine Al_2_O_3_ particles may represent a health risk. Al is considered a neurotoxin. In a previous study we demonstrated that peripheral administration of Al_2_O_3_ nanoparticles (NPs) induced microglia and astrocyte activation in rat hippocampus and cortex [7]. In the present study, mice were exposed to Al_2_O_3_ NPs through a whole-body exposure system, which mimics the actual exposure of suspended Al-containing ultrafine particles in air.

Stress is associated with the pathophysiology of emotional disorder, and women are more vulnerable to anxiety and depression in stress responses [8]. Numerous studies have explored the potential sex differentiation mechanisms; and studies have shown that glucocorticoids are released by the hypothalamus-pituitary-adrenals (HPA) axis more rapidly and intensively in stress responses in females than in males [9]. Hormones influence brain systems and lead to the gender differences of depression [8]. Exposure to environmental chemicals could induce brain dysfunction and depression-like alterations. A number of behavioral effects have been attributed to airborne PMs, including decreased spatial learning capability, spontaneous locomotion induced by diesel exhaust particles [10,11], provoked depression-like responses induced by fine particulate matter (PM_2.5_) coupled with light at night [12], and depression-like behavior induced by zinc oxide NPs [13]. However, gender differentiation was not noticed in those studies. Several studies have focused on association of behavior and the prenatal exposure to environmental PMs, which suggested a potential sex-differentiation. Prenatal exposure to urban air NPs caused depression-like responses in male offspring of mice [14]. Allen *et al.* [15] reported that ultrafine PMs induce autism-like alterations only in male offspring when exposed at an early postnatal stage. The potential mechanisms include increase of glia activation and enhanced hippocampal glutamate in male mice induced by the small particles existing in ambient environment. 

The prefrontal cortex (PFC) plays an important role in several stress-related neuropsychiatric illnesses, including major depressive disorder. Reductions of volume in PFC were found in both bipolar and unipolar depressions [16,17], which are associated with glial loss and neuronal atrophy [18]. Decreased cortical glutamate and glial functions were involved in depression [19]. Bipolar disorders, including both depression and anxiety-like behavior, are observed after lesion formation in PFC [20].

To better understand the paradigm of sex-differentiated response to Al_2_O_3_ NPs, the present study evaluated mice behavior through open field test and forced swim tests as well as aluminum burden, gene expression and metabolites in cerebral cortex of mice administered by respiratory inhalation of Al_2_O_3_ NPs, which mimic the ambient exposure route, to help elucidate the sex-dependent response to environmental stress. 

## 2. Methods

### 2.1. Nanomaterial and Animals

Al_2_O_3_ NPs with an average diameter of 40 nm and >99.8% purity were purchased from Plasmachem GmbH (Berlin, Germany). A total of 20 male and 20 female ICR mice (20–22 g) were purchased from Shanghai SLRC Laboratory Animal Co. Ltd. (Shanghai, China). 

### 2.2. Animal Treatment

Animals were treated humanely, maintained, and used in accordance with Guidelines of Committee on Animal Use and Care of Southeast University. The entire body exposure chamber was fitted with extensive air quality monitoring equipment and an aerosol generator (Beijing HuiRongHe Technology Co., Ltd, Beijing, China). Mice were divided into four groups (with 10 mice in each group), namely male control, female control, male Al_2_O_3_ NP treatment group, and female Al_2_O_3_ NP treatment group. Mice were housed five per polycarbonate cage on corncob bedding with *ad libitum* access to food and water. Exposure was carried out in two whole-body exposure chambers at the same time; the treatment group received Al_2_O_3_ NPs, and the control received HEPA-filtered clean air at the same flow rate as the treatment group. Mice were placed in each chamber from 9 a.m. to 3 p.m. per day for 28 consecutive days. Light cycles were set on a 12/12 h light/dark cycle. The light cycle was maintained from 7 a.m. to 7 p.m. The mean concentrations of Al_2_O_3_ NPs were 0.5 mg/m^3^, and temperature in the chambers was set to 22.5 °C.

### 2.3. Open-Field Tests

Emotional behavior of mice was determined using open field test (OFT) at 3 p.m. after 14 or 28 days Al_2_O_3_ NP treatment. Animals were individually placed in an acrylic box (40 cm × 40 cm) within a soundproof box and then placed on the apparatus for 3 min. After the test, the mice were returned to their home cages. The open field was cleaned between tests. Behavior was recorded and analyzed with video tracking software (Ethovision XT, Noldus Information Technology, Wageningen, The Netherlands). Parameters including entries from periphery to central zones (20 cm × 20 cm), duration in these two zones, speed, mobile and immobile durations, and total distance moved were analyzed.

### 2.4. Forced Swimming Test

Depression-like behavior of mice was determined by a forced swimming test (FST), which started 5 min after the end of OFT on the 28th day of Al_2_O_3_ NPs treatment. Each mouse was placed in a cylindrical tank with a diameter of 19 cm and height of 30 cm. The tank was filled with tap water to the height of 25 cm and the temperature was maintained at 24 ± 1 °C. Mice are considered immobile if they are floating and their hind limbs appear immobile, with only small visible movements of the forepaws to keep their head above water [21]. The behavior was recorded with a video camera for 5 min. The total immobility duration was recorded by an observer blinded to the experiment design. 

### 2.5. RNA Isolation and Quantitative Real-Time PCR Assay

Six mice per group were decapitated after ether anesthesia 24 h after the FST. The whole cortical tissue of brain was collected and kept in liquid nitrogen. Half of the cerebral cortex was prepared for total RNA extraction and the other half was kept for metabolomics analysis. The total RNA of brain tissues was extracted using a PureLink^®^ RNA Mini Kit (Invitrogen, Carlsbad, CA, USA) according to the manufacturer’s protocol. The concentration of total RNA was determined by measuring the absorbance at 260 nm using a Nanodrop 2000c spectrophotometer (Thermo Scientific, Waltham, MA, USA). cDNA synthesis for coding genes was performed with 1 μg of total RNA according to the manufacturer’s instruction (Toyobo, Osaka, Japan).

The modulated genes were screened based on previous mRNA microarray data (unpublished; Tables S1 and S2). The mRNA levels were determined by quantitative real-time PCR analysis (qRT-PCR) on a Quant Studio 6 Flex system (Applied Biosystems, Life Technologies, Carlsbad, CA, USA) using SYBR^®^ Green Realtime PCR Master Mix-Plus (Toyobo, Osaka, Japan) in accordance with the manufacturer’s protocol. Primers were designed for the modulated genes provided in supplemental Table S3. All experiments were performed in triplicates. The mRNA levels provided were normalized to cyclophilin A.

### 2.6. Pathological Analysis

Four mice per group were euthanized with ether anesthesia 24 h after the forced swimming test. Lungs and brains of mice were rapidly removed. A piece of lung and half brain tissues were fixed with 4% paraformaldehyde (PFA) overnight at 4 °C, embedded in paraffin, serially sectioned (5 μm), and then mounted on silane-covered slides. The other half brain and a piece of lung tissues were kept in liquid nitrogen for aluminum burden analysis. After dewaxing, the sections selected at corresponding layers from each mouse were stained with hematoxylin and eosin (H&E). The severity of pathological lesions was scored according to Szapiel’s method [22] as follow: 1= no appartent alveolitis;2 = mild alveolitis (with pulmonary interstitial edema, inflammatory cell infiltration, and alveolar septum thickening, with lesions only local or limited to the subpleural area, which do not exceed 20% of the lung); 3 = moderate alveolitis (with the subpleural area more obvious, with the involved area more than 20% but less than 50% of the lung); 4 = severe alveolitis (with lesions more than 50% of the lung, with inflammatory cells inside the alveolar cavity and consolidation changes). Double-blind examination of alveolitis was conducted by an experienced pathologist using a light microscope, and the alveolitis scores were presented as a mean for each sample.

### 2.7. Aluminum Burden

The cortex and midbrain were separated from the half brain of mice. Then, the brain and lung samples were weighed.Approximately 0.05 g of each sample was digested with HNO3 in a boiling water bath for 3 h. The aluminum burdens in different brain regions were quantified using an inductively coupled plasma mass spectrometry (ICP-MS, Agilent 7700, Santa Clara, CA, USA).

### 2.8. Metabolomics Analysis

Metabolomics analysis was performed according to the report of Xu *et al.* [23]. Briefly, to each mouse cortex sample was added 50% methanol (1 mL) and it was homogenized, and the supernatant was obtained after centrifugation at 12,000 rpm for 15 min. About 350 μL of the supernatant was transferred into a 2 mL vial and blow-dried by nitrogen. Afterward, 80 μL of methoxyamine was added and reacted for 2 h at 37 °C. Finally, 100 μL of bis (trimethylsilyl)trifluoroacetamide (BSTFA) reagent (containing 1% TMCS) was added to the mixture and reacted for 60 min at 70 °C. The sample was cooled down to room temperature and then analyzed by GC/TOF/MS (Agilent 7890 coupled with a Pegasus 4D TOF). 1 μL of the sample was injected in split less mode at a gas flow rate of 1 mL/min. The program was set at 80 °C for 0.2 min, raised to 180 °C at a rate of 10 °C/min, then to 240 °C at a rate of 5 °C/min, and finally to 290 °C at a rate of 20 °C/min. 

### 2.9. Data Analysis

Data are presented as the mean ± standard error of the mean. Statistical analyses were conducted using SPSS 16.0. Two-way ANOVA followed by Student’s *t*-test was used to compare the parameters of aluminum burden and fold induction of gene expression. Two-way repeated measures ANOVA, with sex as independent factors and duration of exposure as within-subjects factor was used to compare the parameters of behavioral studies on treatment day 14 and 28. Kruskal-Wallis test was used to analyze the ranked data of pulmonary alveolitis. The significance was set as *p* < 0.05. Two-way ANOVA followed by Bonferroni *post hoc* comparisons was performed in metabolomics analysis. The cut-off for metabolomics analysis is fold change >1.5 and *p* < 0.05.

## 3. Results

### 3.1. Female Mice Demonstrated Depressive-Like Behavior Following Al_2_O_3_ NP Exposure.

**Figure 1:**
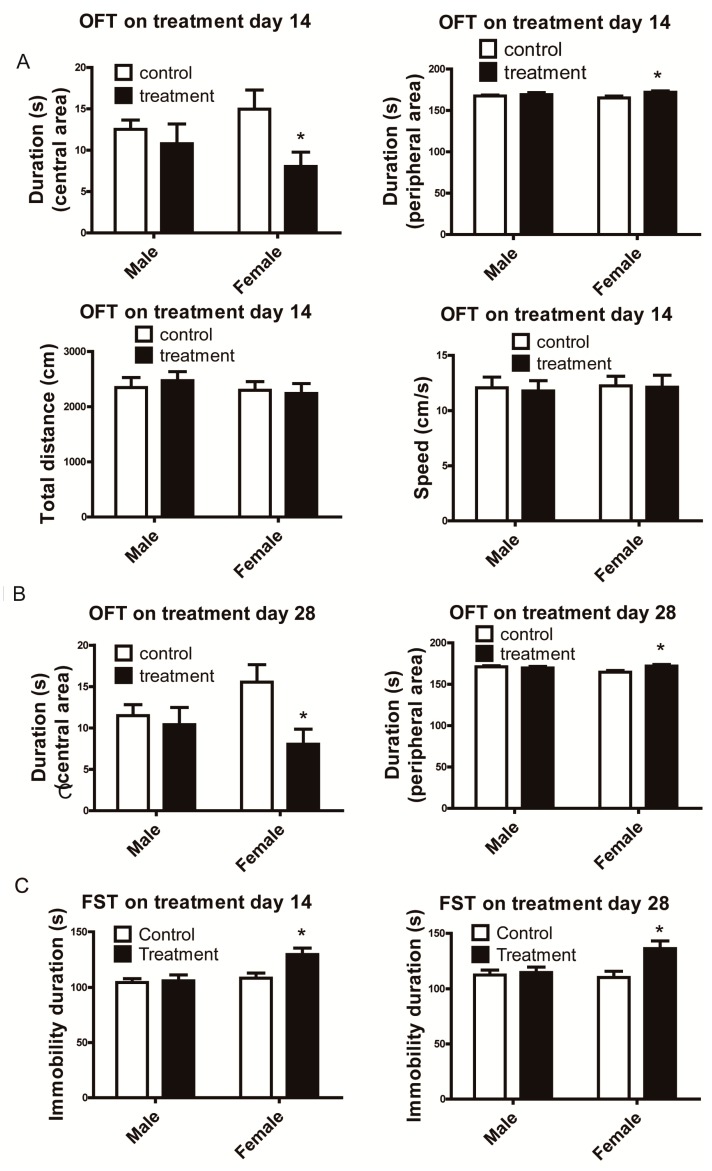
Emotional behavioral tests of mice treated by Al_2_O_3_ NPs. (**A**): female mice showed less duration in central zone and increased duration in periphery zone in OFT on treatment day 14, (**B**): On the treatment day 28, results were consistent with day 14, (**C**): female mice showed increased immobile duration in FST on treatment day 14 and 28. * *p* < 0.05, compared with control of corresponding sex, n = 10.

In OFT, Al_2_O_3_ NPs treated female mice exhibited less duration in the central zone and enhanced duration in peripheral zone which implied less exploring behavior and spontaneous locomotion [13] compared with female control after 14 days-treatment (*t* = 7.098, df = 9, *p* < 0.00001) (Figure 1A) and 28 days of treatment (*t* = 9.777, df = 9, *p* < 0.00001) (Figure 1B). 

Male mice did not show significant differences between control and treatment (Figure 1A,B). Female mice exposed to Al_2_O_3_ NPs had higher immobility time during the 5 min FST than female control after 14 (*t* = 8.692, df = 9, *p* < 0.00001) or 28 (*t* = 7.558, df = 9, *p* < 0.00001) days of treatment (Figure 1C). Consistent with the OFT, mice treated with Al_2_O_3_ NPs did not show any behavioral alterations in FST (Figure 1C).The interaction effects between sex and Al_2_O_3_ NPs treatment were showed in central area duration (F = 14.17, *p* = 0.0008 on day 14; F = 37.56, *p* < 0.0001 on day 28, df = 9), peripheral area duration (F = 14.17 *p* = 0.0008 on day 14; F = 37.56, *p* < 0.0001 on day 28, df = 9) and immobility duration (F = 32.39, *p* < 0.0001 on day 14; F = 25.18, *p* < 0.0001 on day 28, df = 9) following 14 or 28 days of treatment. 

For OFT and FST, the data following 14 and 28 days of treatment were compared through two-way repeated measures ANOVA, with sex as independent factors and duration of exposure as within-subjects factor. However, there were no significant differences between 14- and 28-day observations (for OFT central area duration F = 1.780, *p* = 0.1929; peripheral area duration F = 1.780, *p* = 0.1929; for FST F = 0.2518, *p* = 0.6197).

### 3.2. Aluminum Burden Varied in Brain and Lung Tissues of Mice

**Figure 2:**
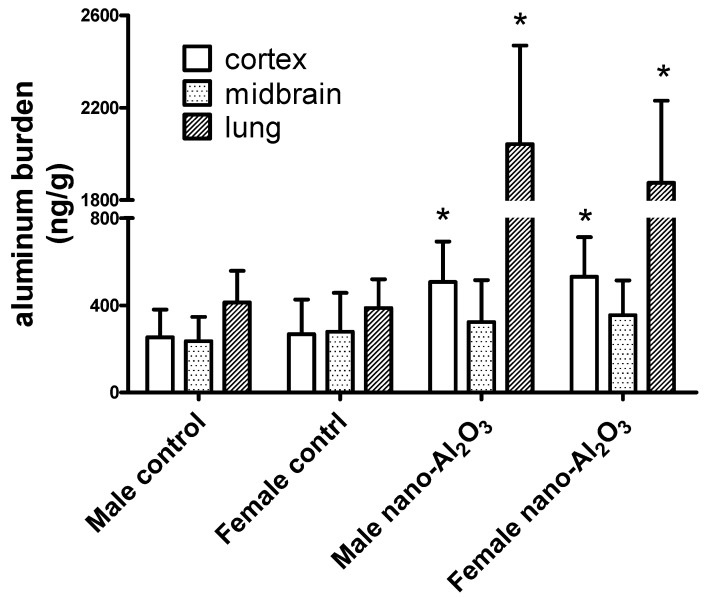
Aluminum burden in cortex, midbrain, and lung tissues of mice. * *p* < 0.05, compared with the corresponding sex control, n = 4.

Results in Figure 2 demonstrate that the aluminum burden in the lung tissues of Al_2_O_3_NP-treated mice significantly increased compared with their corresponding sex control (for male mice, *t* = 18.290, df = 3, *p* < 0.00001; for female mice, *t* = 18.700, df = 3, *p* < 0.00001). 

Similar trends of enhancement were noted in the cortex but not midbrain (for male mice, *t* = 2.845, df = 3, *p* = 0.01125; for female mice, *t* = 3.316, df = 3, *p* = 0.0042). The aluminum concentrations between male and female in the same tissue were also compared. No significant sex-dependent differences were observed, therefore, the increased aluminum burden in cortex of mice could not account for the differences of neurobehaviors between male and female mice. More investigations on the underlying mechanisms in mice brains are required.

### 3.3. Pathological Alterations of Lung but not Brain Tissues of Mice Were Observed

**Figure 3:**
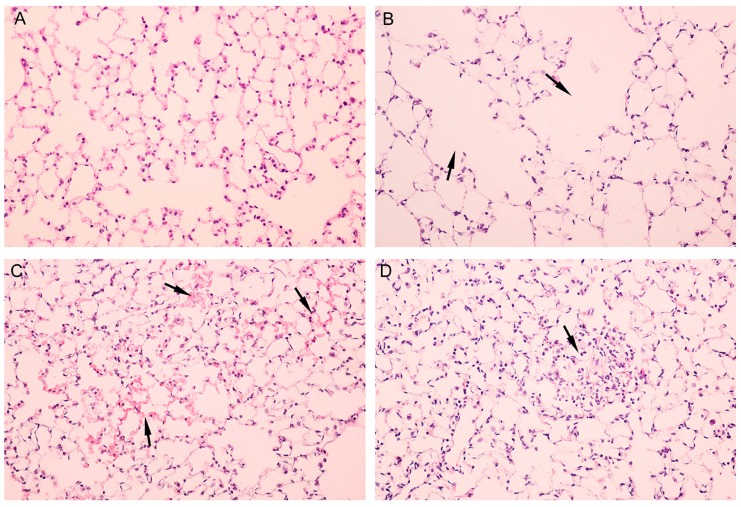
Pathology of mice lung tissues following Al_2_O_3_ treatment. (**A**): control; (**B**): arrows showed ruptured alveolar walls and fusion of pulmonary alveoli; (**C**): arrows showed pneumorrhagia in lung tissues; (**D**): arrows demonstrated lymphocyte infiltration in lung tissues.

Lesions in lung tissues were scored (seen in Table 1). Both male and female mice showed mild to moderate alveolitis (2.25 ± 0.50 for male mice, 2.5 ± 0.58 for female mice) following Al_2_O_3_ NP exposure compared with corresponding sex control (for male mice *p* < 0.0001; for female mice *p* < 0.0001). No differences were observed between male and female Al_2_O_3_NP-treated mice. Figure 3A shows lung tissue of control group. Figure 3B shows ruptured alveolar walls and fusion of pulmonary alveoli. Figure 3C demonstrates pneumorrhagia in lung tissues. Figure 3D shows lymphocyte infiltration in lung tissues. No significant pathological alterations were observed in the brain cortex. 

**Table 1:** Severity of lesions in lung tissues response to nano Al_2_O_3_ treatment.

Group	Lesion Severity Grade				Average Severity Grade
	1	2	3	4	
male control	4				1
female control	4				1
male nano-Al_2_O_3_		3	1		2.25 ± 0.50 *
female nano-Al_2_O_3_		2	2		2.5 ± 0.58 *

* *p* < 0.05, compared with the corresponding sex control, n = 4.

### 3.4. Expressions of Mental Disorder Involved Genes Were Modulated in a Sex-Dependent Manner

To further elucidate the potential mechanism of sex-dependent neurobehavioral alterations induced by Al_2_O_3_ NPs, the expression levels of mental disorder-related genes were detected by qRT-PCR. The expression levels of 16 genes in mice cerebral cortex were validated by qRT-PCR (Table 2). Ten genes, including SNAP29, ZDHHC8, GDNF, GNAS, NOS3, GRIN2A, DGKH, MT2A, ACNA1G and KCNQ showed interaction effects between sex and Al_2_O_3_ NP treatment (*p* < 0.05).We noticed that these sex-differentially expressed genes were involved with neurotransmitter, phosphatidic acid synthesis, and voltage-gated ion channels, which occupied important positions in neural functions. Most of the predicted downregulated gene expressions in the mice cortex were consistent with the array data. These genes were significantly downregulated in both male and female brain tissues. The expression levels of DGKH, CACNA19, MT2A, and KCNQ2 in male mice were enhanced (*p* < 0.05), whereasGRIN2A was not significantly modulated. In female mice cerebral cortex, the expressions of MT2A and GRIN2A were upregulated (*p* < 0.05), whereas the levels of DGKH, CACNA1G, and KCNQ2 were significantly downregulated (*p* < 0.05).

### 3.5. Metabolomics Analysis Explored the Potential Role of Glutamate

Bonferroni post hoc comparisons were used to identify the key small molecules alterations. A total of 10 different expression metabolites were identified in the cortex of male mice including seven upregulated and three downregulated; 12 modulated metabolites were found in the cortex of female mice including eight upregulated and four downregulated. Notably, two glutamate metabolism-related molecules, l-glutamic acid and pyroglutamic acid, decreased in female tissues, which showed an inconsistent trend with male. The list of modulated metabolites, their corresponding human metabolome database (HMDB) ID and fold change are shown in Table 3.

**Table 2:** Fold induction of modulated genes in mice cortex.

	Control		Treatment							
	Male	Female	Male	Female	F Value			*p* Value		
	Fold Change	Fold Change	Fold Change	Fold Change	Treatment	Sex	Interaction	Treatment	Sex	Interaction
SNAP29	1.03 ± 0.10	1.02 ± 0.12	0.95 ± 0.11	0.62±0.14 *^,#^	24.64	12.36	10.95 ^&^	<0.0001	0.0022	0.0035
UFD1L	1.01 ± 0.07	1.02 ± 0.06	0.61 ± 0.08 *	0.65±0.08 *	167.00	0.7042	0.2535	<0.0001	0.4113	0.6201
ZDHHC8	1.04 ± 0.05	1.05 ± 0.07	0.95 ± 0.07 *	0.58±0.06 *^,#^	131.40	41.09	46.23 ^&^	<0.0001	<0.0001	<0.0001
PDLIM5	1.01 ± 0.08	1.05 ± 0.10	0.71 ± 0.10 *	0.82±0.14 *	36.64	2.935	0.639	<0.0001	0.1022	0.4334
HSPA1A	1.06 ± 0.10	0.99 ± 0.07	0.54 ± 0.11 *	0.56±0.09 *	154.30	0.4273	1.385	<0.0001	0.5207	0.2531
PTEN	0.99 ± 0.08	1.03 ± 0.09	0.63 ± 0.11 *	0.67±0.12 *	75.86	0.94	0.00	<0.0001	0.3447	1.0000
GDNF	1.01 ± 0.07	0.98 ± 0.05	0.65 ± 0.06 *	0.32±0.14 *^,#^	204.00	25.41	17.65 ^&^	<0.0001	<0.0001	0.0004
SOD2	0.97 ± 0.06	0.99 ± 0.08	0.67 ± 0.12 *	0.56±0.07 *	109.10	1.66	3.46	<0.0001	0.2125	0.0776
GNAS	1.01 ± 0.09	1.06 ± 0.12	0.88 ± 0.15	0.70±0.14 *^,#^	22.30	1.57	4.91 ^&^	0.0001	0.2247	0.0384
NOS3	0.96 ± 0.11	1.00 ± 0.04	0.71 ± 0.08 *	0.93±0.15 ^#^	14.42	9.52	4.56 ^&^	0.0011	0.0058	0.0452
DLST	1.01 ± 0.04	0.97 ± 0.06	0.91 ± 0.12	0.87±0.06	10.34	1.66	0.00	0.0043	0.2130	1.0000
GRIN2A	0.97 ± 0.08	1.01 ± 0.06	1.10 ± 0.18	2.78±0.17 *^,#^	423.70	317.70	288.20 ^&^	<0.0001	<0.0001	<0.0001
DGKH	1.01 ± 0.04	1.06 ± 0.09	2.14 ± 0.09 *	0.72±0.06 *^,#^	175.00	526.21	605.94 ^&^	<0.0001	<0.0001	<0.0001
MT2A	1.01 ± 0.07	1.02 ± 0.15	1.98 ± 0.07 *	2.34±0.13 *^,#^	639.50	16.70	14.94 ^&^	0.0010	0.0006	<0.0001
CACNA1G	1.04 ± 0.07	1.03 ± 0.06	2.77 ± 0.13 *	0.75±0.12 *^,#^	516.75	938.68	887.50 ^&^	<0.0001	<0.0001	<0.0001
KCNQ	1.00 ± 0.04	0.97 ± 0.08	2.86 ± 0.10 *	0.72±0.11 *^,#^	317.03	621.19	609.14 ^&^	<0.0001	<0.0001	<0.0001

* *p* < 0.05, compared with corresponding sex control, ^#^ *p* < 0.05, compared with male Al_2_O_3_ NP treated mice, ^&^ *p* < 0.05, sex and Al_2_O_3_ NPs treatment showed interaction effect, df = 5.

**Table 3:** Metabolomics analysis of Al_2_O_3_NP-treated mice cortex.

Metabolite	HMDB ID	Fold Change			Male Al_2_O_3_ NPs *vs.* Male Control		Female Al_2_O_3_ NPs *vs.* Male Control	
		Male Al_2_O_3_ NPs	Female Control	Female Al_2_O_3_ NPs	F Value	*p* Value	F Value	*p* Value
L-Serine	HMDB00187	1.98	1.03	4.26 ^#^	13.63	0.002	53.54	<0.0001	
Dihydroxy acetone Phosphate	HMDB01473	-	0.83	2.97 ^#^	0.43	0.5261	14.9	0.0017	
Inosine	HMDB00195	2.05	1.14	2.61	12.68	0.0022	13.01	0.0019	
Prostaglandin E2	HMDB01220	2.43	1.27	4.02	22.31	0.0001	49.69	<0.0001	
Acetyl-L-Carnitine	HMDB00201	2.37	0.96	3.86	28.74	<0.0001	37.21	<0.0001	
Pyroglutamic Acid	HMDB00267	2.53	0.89	0.52 ^#^	32.57	<0.0001	24.52	<0.0001	
Riboflavin	HMDB00244	3.83	1.07	2.18	36.53	<0.0001	27.73	<0.0001	
Inosine 5-Monophosphate	HMDB00175	1.89	0.93	1.68	11.42	0.0026	9.52	0.0058	
l-Glutamic Acid	HMDB00148	-	1.08	0.17 ^#^	1.69	0.2125	103.89	<0.0001	
7b-Hydroxycholesterol	HMDB06119	0.48	0.77	-	28.49	<0.0001	0.89	0.4213	
2-Hydroxyestrone	HMDB00343	-	0.98	0.48	0.25	0.6178	41.25	<0.0001	
Dl-Glyceraldehyde	HMDB01051	0.3	1.06	0.46	59.77	<0.0001	38.56	<0.0001	
Petroselinic Acid	HMDB02080	0.21	1.18	0.48	75.86	<0.0001	42.59	<0.0001	

^#^ *p* < 0.05, compared with male Al_2_O_3_ NPs treatment, df = 5.

## 4. Discussion

In this study, we observed that respiratory inhalation of Al_2_O_3_NPs increased depression-like behavior only in female mice. Gene expression and metabolomics analysis implied strong modulations of glutamate pathway in a sex-dependent manner. 

Behavioral tests demonstrated decreased spontaneous locomotion, exploring behaviors and increased despair-like behavior in female mice exposed to Al_2_O_3_ NPs. Environmental ultrafine particles were associated with depression-like behavior. The potential mechanisms included oxidative stress, lipid peroxidation, and disruption of electrophysiological processes [13,24]. Differences between genders were not mentioned in previous studies. Prenatal exposure to NPs collected from urban air also induces depression-like responses, but only in male offspring [15]. The variation of chemicals and exposure time window may affect behavioral outcomes. In the present study, exposure duration did not show interaction with behavioral alterations. Further studies are still required to explore whether a longer exposure could aggravate the damages of behaviors induced by Al_2_O_3_ treatment.

Aluminum burden in the cortex and midbrain of mice was examined. Significant increases were noted in the lung and cortex tissues of Al_2_O_3_NP-treated mice of both sexes, suggesting that aluminum particles could reach brain tissues through respiratory exposure. However, this enhancement of aluminum burden in the cortex region could not explain the sex-dependent differences in depression-like behavior of mice. These data implied that aluminum existing in cortex could trigger a different profile of biological responses in male and female, thus warranting investigation of the underlying molecular correlations. Furthermore, we assessed the gene expression levels and alterations of metabolites to explore the potential sex-dependent depression mechanism.

The voltage-sensitive calcium channel, which is encoded by CACNA1G, mediates the entry of calcium ions into excitable cells and is also involved in various calcium-dependent processes such as neurotransmitter release, cell division, and cell death. KCNQ2 encodes subfamily Q of potassium-voltage-gated channel, which plays a critical role in the regulation of neuronal excitability. Voltage-gated channels contribute to the regulation of diverse neuronal functions through elevating intracellular ion concentrations [25,26]. The defects in the expression or modulation of presynaptic ion channels may result in neural network dysfunction. The heterozygous rol/+ rolling mice showed less immobility time than the wild type (+/+), which is attributed to the presence of calcium channel subunit mutation [21]. We found that two voltage-gated ion channel-related genes, CACNA1G and KCNQ2, were differently regulated in male and female mice exposed to Al_2_O_3_ NPs. This implied that down-regulation of voltage-gated ion channels is associated with depressive-like behavior in female mice.

DGKH encodes a member of the diacylglycerol kinase enzyme family and plays a key role in promoting cell growth. Variation in this gene is associated with bipolar disorder [27] and unipolar depression [28]. Results showed a sex-dependent regulation in DGKH expression. 

Metabolomics analysis help to understand the endogenous small molecules alteration induced by Al_2_O_3_ NPs, and the results also suggest effects in a sex-dependent manner. l-Glutamic acid, one of the most abundant fast excitatory neurotransmitters in the mammalian nervous system, is suggested to play a key role in depression development. Once released by presynaptic cells, glutamic acid binds and activates glutamate receptors including NMDA receptor. Increased expression of NMDA receptor was observed in major depression patients [29,30]. Our qRT-PCR results showed significantly upregulated GRIN2A in Al_2_O_3_ NPs treated female mice cortex, which is a glutamate (NMDA) receptor subunit-encoding gene. Pyroglutamic acid is a cyclized derivative of l-glutamic acid. Altered pyroglutamic levels imply disruption of glutamine metabolism. Interestingly, down-regulation of l-glutamic acid was observed only in female mice cortex, and pyroglutamic acid increased in male but decreased in female mice cortex treated with Al_2_O_3_ NPs. Depressive disorder is associated with altered function of excitatory neurotransmitter glutamate; reduced glutamate levels were observed in depressive patients or in animal models [31,32]. Up-regulation of GRIN2A was observed in dorsolateral prefrontal cortex of female major depressive disorder cases, which is consistent with our results [30]. In the present study, differential regulation of glutamate pathway-related metabolites was observed in a sex-dependent manner. In addition to upregulation of glutamate receptor subunit GRIN2A in female mice, our results strongly suggested that the glutamate pathway was involved in the Al_2_O_3_NP-induced depression-like behavior in female mice.

One of the limitations in the present study was that only the filtered clean air control was set to observe the toxic effects of exposure to Al_2_O_3_ NPs. Thus, current results could not elucidate whether the damages observed as a result of chemical or mechanical effects of exposure. One more group exposed to aluminum particles would provide insight to determine whether the phenotype observed in this study resulted from exposure to aluminum or the nanoparticles themselves. The other limitation of our study was that whole cortex tissue but not PFC were used for the gene expression, metabolomics and aluminum burden analysis. Due to the small volume of mice brains, PFC tissues are not enough for these analyses. Thus, more mice and better experimental design should be provided in the further studies.

## 5. Conclusions

In brief, metabolomics analysis coupled with the modulated gene expression in mice cerebral cortex explored sex-dependent` alterations in mice treated with Al_2_O_3_ NPs, including the neural behavior, voltage-gate ion channels, neural transmitter, and small molecule metabolisms. These results could partly explain the sex-dependent response to environmental stress and prompt the importance of sexual dimorphism in environmental toxin research.

## Supplementary Files

Supplementary File 1
