# Supplementary material for: Sex-Dependent Depression-Like Behavior Induced by Respiratory Administration of Aluminum Oxide Nanoparticles
Source: Int J Environ Res Public Health. 2015 Dec 9;12(12):15692–705. doi: 10.3390/ijerph121215011 (PMC4690947; doi:10.3390/ijerph121215011)
Supplement: Supplementary File 1 [file ijerph-12-15011-s001.pdf]

Supplementary Information

Sex-Dependent Depression-Like Behavior Induced by Respiratory Administration of Aluminum Oxide Nanoparticles

Table S1. Modulated global expression genes in HBE involved in mental disorders.

Disease Term	Involved Genes
Schizophrenia	SNAP29, UFD1L, ZDHHC8, PDLIM5, GRIN2A, GNAS, HSPA1A, CHRFAM7A, GDNF, SOD2
Alzheimer's Disease	DLST, NOS3, HSPA1A, CHRFAM7A, PTEN, SOD2
Bipolar disorder	GRIN2A, NOS3, DGKH, CHRFAM7A
Parkinson's disease	NOS3, HSPA1A, SOD2
Dementia	NOS3, HSPA1A
Schizophrenia	ZDHHC8, PDLIM5
Depressive disorder	HSPA1A, CHRFAM7A

Table S2. Functions and modulation of mental disorder related genes in HBE predicted by microarray.

Gene Symbol	Fold Change	Regulation	Product
SNAP29	3.20	down	synaptosomal-associated protein 29
UFD1L	2.95	down	ubiquitin fusion degradation 1 like (yeast)
ZDHHC8	3.24	down	probable palmitoyltransferase ZDHHC8 isoform 2
PDLIM5	1.57	down	PDZ and LIM domain protein 5 isoform e
HSPA1A	2.47	down	heat shock 70 kDa protein 1A/1B
PTEN	3.70	down	phosphatidylinositol 3,4,5-trisphosphate 3-phosphatase and dual-specificity protein phosphatase PTEN
GDNF	2.12	down	glial cell derived neurotrophic factor
SOD2	2.10	down	superoxide dismutase [Mn], mitochondrial isoform A precursor
GNAS	2.43	down	protein ALEX isoform f
NOS3	1.51	down	nitric oxide synthase, endothelial isoform 1
DLST	2.056	down	dihydrolipoyllysine-residue succinyltransferase component of 2-oxoglutarate dehydrogenase complex
GRIN2A	2.52	up	glutamate [NMDA] receptor subunit epsilon-1 isoform 2 precursor
DGKH	2.49	up	diacylglycerol kinase eta isoform 1
MT2A	2.28	up	metallothionein-2
CACNA1G	1.58	up	voltage-dependent T-type calcium channel subunit alpha-1G isoform 14
KCNQ2	1.55	up	potassium voltage-gated channel subfamily KQT member 2 isoform c

Table S3. Primer sequences for qRT-PCR analysis.

Gene Symbol	Forward Primer	Reverse Primer
SNAP29	5'- TTCGACGATGACGTGGAAGAG-3'	5'- GGTACTGCTGCCTGTCAATGG-3'
UFD1L	5'-CCTCAGCCCTCGATCAACTC-3'	5'-CAGTACACCGCAGTGTGTCAT-3'
ZDHHC8	5'-CCCTCTCCTGTGCGCTATG-3'	5'-TCGCCAAAGAGTGAGTCAGTC-3'
PDLIM5	5'-AAAGGAAAAGATACCCCTTCACG-3'	5'-GTGCTGAAACTTCATGGTGCC-3'
GRIN2A	5'-ACGTGACAGAACGCGAACTT-3'	5'-TCAGTGCGGTTCATCAATAACG-3'
HSPA1A	5'-TGGTGCAGTCCGACATGAAG-3'	5'-GCTGAGAGTCGTTGAAGTAGGC-3'
DGKH	5'-CACCTTCTGTAACGTGTGCAG-3'	5'-CCATCCTCGTCCTCTATGATGTC-3'
PTEN	5'-TGGATTCGACTTAGACTTGACCT-3'	5'-GCGGTGTCATAATGTCTCTCAG-3'
GDNF	5'-TCCAACTGGGGGTCTACGG-3'	5'-GCCACGACATCCCATAACTTCAT-3'
GNAS	5'-CAGAGCCTCCATTGGGGTC-3'	5'-GCTTCTCGCTCAACTGGGG-3'
NOS3	5'-GGCTGGGTTTAGGGCTGTG-3'	5'-CTGAGGGTGTCGTAGGTGATG-3'
MT2A	5'-CGCCATGGATCCCAACTG-3'	5'-CCTGAAATTGAGTGACCAGAGAAA-3'
CACNA1G	5'-TGTCTCCGCACGGTCTGTAA-3'	5'-AGATACCCAAAGCGACCATCTT-3'
KCNQ2	5'-TGACTGCCTGGTACATTGGC-3'	5'-CTCTTGGACTTTCAGGGCAAA-3'

© 2015 by the authors; licensee MDPI, Basel, Switzerland. This article is an open access article distributed under the terms and conditions of the Creative Commons Attribution license (http://creativecommons.org/licenses/by/4.0/).
